# N‑Sulfated Heparan Sulfate Promotes Reelin Signaling as a Co-receptor

**DOI:** 10.1021/jacs.5c15573

**Published:** 2025-12-08

**Authors:** Lin Pan, Xuehong Song, Guowei Su, Lauren A Gandy, Biqin Fang, Mason Buttaci, James Gibson, Ke Xia, Fuming Zhang, Jian Liu, Lianchun Wang, Sally Temple, Chunyu Wang

**Affiliations:** † Center for Biotechnology and Interdisciplinary Studies, 8024 Rensselaer Polytechnic Institute, Troy, New York 12180, United States; ‡ Department of Chemistry and Chemical Biology, Rensselaer Polytechnic Institute, Troy, New York 12180, United States; § Department of Molecular Pharmacology and Physiology, Byrd Alzheimer’s Center and Research Institute, 33697 Morsani College of Medicine, University of South Florida, Tampa, Florida 33612, United States; ∥ Glycan Therapeutics, Raleigh, North Carolina 27606, United States; ⊥ 347511 Neural Stem Cell Institute, Albany, New York 12208, United States; ¶ Division of Chemical Biology and Medicinal Chemistry, Eshelman School of Pharmacy, University of North Carolina, Chapel Hill, North Carolina 27599, United States

## Abstract

Heparan sulfate (HS) plays a central role in signal transduction, while Reelin is an essential signaling protein in both the developing and adult brain. A Reelin COLBOS variant was recently discovered with enhanced HS binding and resilience against autosomal dominant Alzheimer’s disease (ADAD), underscoring the importance of Reelin–HS interactions. However, the glycan determinants of Reelin–HS interactions have not been well-characterized, which we systematically investigated here. Surface plasmon resonance (SPR) showed that full length Reelin binds HS with high affinity (*K*_D_ = 17 ± 5 nM), which is enhanced by the COLBOS variant (*K*_D_ = 10 ± 2 nM). Competition SPR and glycan array studies further revealed that HS N-sulfation is critical for Reelin–HS binding, consistent with Haddock modeling. In cell surface binding assays, heparinase treatment, which degrades HS, or the knockout of a key HS N-sulfation enzyme (NDST1) significantly reduced Reelin attachment. Functionally, a cellular split-luciferase assay showed that heparinase treatment or adding heparin in culture medium reduces Reelin-induced ApoER2 dimerization, demonstrating that HS is a coreceptor for Reelin receptor activation. In contrast, N-desulfated heparin does not inhibit Reelin receptor dimerization. Our work establishes HS as a coreceptor for Reelin signaling and N-sulfation as a key glycan determinant of Reelin–HS recognition. Our work provides mechanistic insights into diverse neurodevelopmental and neurodegenerative diseases associated with Reelin signaling and suggests novel therapeutic strategies targeting HS sulfation.

## Graphical abstract

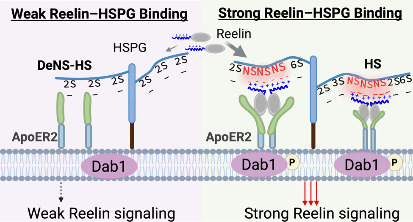

Reelin is a large, secreted extracellular glycoprotein^1^ (Figure 1B), which binds to receptors such as ApoER2 (LRP8) and VLDLR to activate intracellular signaling cascades.^2^ Disruptions in Reelin signaling are linked to many neuropsychiatric disorders, including depression, schizophrenia, autism spectrum disorders, epilepsy, and recently AD.^3,4^ In 2023, Quiroz et al. reported the world’s second case of extreme resilience to autosomal dominant AD (ADAD): a patient with a RELN-COLBOS (H3447R) genetic variant was protected against dementia despite carrying an ADAD mutation, PSEN1 E280A.^5^ Interestingly, H3447R does not affect Reelin binding to ApoER2 or VLDLR. Quiroz et al. demonstrated that the H3447R promotes Reelin binding to HS, likely by increasing electrostatic interactions between the positive charges on Reelin (e.g., arginine side chains) and negatively charged sulfo groups on HS. These intriguing data from the COLBOS genetic variant suggest that heparan sulfate may play an important role in the Reelin signaling pathway.

**Figure 1:**
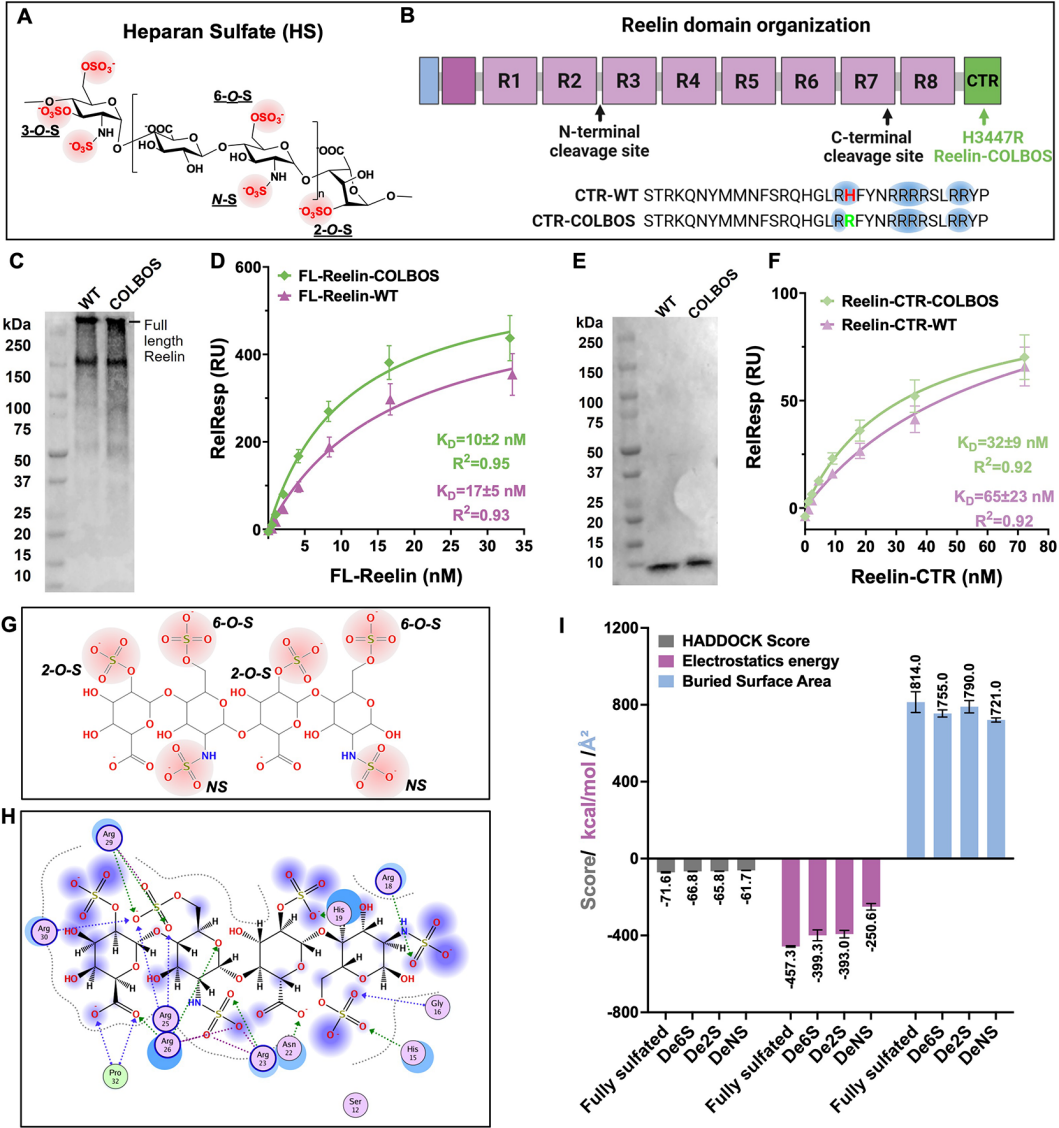
Reelin-heparin binding is driven by specific sulfation patterns. (A) HS representative structures, the negative charge highlighted in red. (B) Reelin domain organization, the CTR positive charge highlighted in blue. (C) Purified FL-Reelin was validated by Western-Blot. (D) Steady state SPR analysis of FL-Reelin binding to heparin. (E) Purified Reelin-CTR was validated by Western-Blot. (F) Steady state SPR analysis of Reelin-CTR binding to heparin. (G) Canonical HS tetrasaccharide used for docking simulations. (H) Docking model: HS tetrasaccharide binds Reelin-CTR-WT via sulfate–Arg/Lys interactions. (I) Quantitative comparison of HADDOCK scores, electrostatic interaction energy (kcal/mol), and buried surface area (Å^2^) for the fully sulfated and desulfated HS variants. De6S, 6-O-S desulfated tetrasaccharide, De2S, 2-O-S desulfated tetrasaccharide, DeNS, NS desulfated tetrasaccharide.

Heparan sulfate (HS) is an important class of polyanionic glycosaminoglycans (GAGs), with crucial signaling roles in the central nervous system^6^ such as mediating neuronal migration in development.^7^ Structurally, HS and heparin (HP, a highly sulfated HS analogue) are linear GAGs composed of repeating disaccharide units of *N*-acetylglucosamine (GlcNAc) and uronic acid (either d-glucuronic acid (GlcA) or l-iduronic acid (IdoA)) (Figure 1A). These units are modified by sulfotransferases in the Golgi, starting with *N*-sulfation (NS) of glucosamine, followed by 2-*O*-sulfation (2S) of uronic acids, and finally 6-*O*-sulfation (6S) and 3-*O*-sulfation (3S) of glucosamine, creating diverse sulfation patterns.^8^ Changes in sulfation of HS chains can greatly alter the affinity of HS-binding proteins, with important consequences on cellular signaling and biological function.^9–11^

We first evaluated the binding affinity between Reelin and heparin (a commonly used HS analog). We expressed and purified full-length Reelin (FL-Reelin; ∼400 kDa; Figure 1B) with either the wild-type (FL-Reelin-WT) or COLBOS (FL-Reelin-COLBOS) sequence (Figure 1C; Figure S1A and B) in mammalian cells. The COLBOS variant is in the Reelin C-terminal region (CTR), which plays an important role in heparin binding.^5^ The CTR of Reelin WT (Reelin-CTR-WT) and the COLBOS (Reelin-CTR-COLBOS) variant were expressed in *E. coli* and purified (Figure 1E; Figure S1C–E). Then we performed the SPR analysis on a heparin biochip as described in our previous studies.^10^ Binding affinity was determined using a steady-state affinity equation.^12^ FL-Reelin-WT exhibited a *K*_D_ of 17 ± 5 nM, while the FL-Reelin-COLBOS variant showed an enhanced binding affinity, with a *K*_D_ of 10 ± 2 nM (Figure 1D). The CTR retains high-affinity binding to heparin with a *K*_D_ of 65 ± 23 nM for the Reelin-CTR-WT. As expected, the Reelin-CTR-COLBOS variant displayed enhanced binding, with a *K*_D_ of 32 ± 9 nM (Figure 1F). These data are consistent with previous bilayer interferometry (BLI) and isothermal titration calorimetry (ITC) studies of CTR–heparin interaction.^5^ However, in Quiroz et al.^5^ only studied Reelin-heparin binding with CTR. Here, we have extended the binding studies to FL-Reelin. Because FL-Reelin and the CTR have *K*_D_ values on the same order of magnitude, CTR is likely the major heparin or HS binding site in Reelin.

To dissect the role of individual sulfate groups in HS–Reelin binding, we employed Haddock^13^ to dock Reelin CTR to a canonical HS tetrasaccharide containing two *N*-, two 2-*O*-, and two 6-*O*-sulfate groups (Figure 1G). Targeted removal of each sulfate type yielded three selectively desulfated variants (DeNS, De2S, and De6S), which were docked to the Reelin-CTR to assess their relative binding contributions. Docking analysis showed that clustered sulfate groups form salt bridges and hydrogen bonds with multiple basic residues in the Reelin-CTR, consistent with multivalent electrostatic recognition (Figure. [Fig fig1]H). R18 and R23 in the Reelin-CTR contribute to recognition of N-sulfated heparan sulfate motifs, providing a plausible structural explanation for its preference toward specific HSPG species. A fully sulfated HS tetrasaccharide displayed the strongest complementarity and largest buried surface area, whereas removal of the N-sulfate group caused the greatest loss of electrostatic energy (>+200 kcal/mol), reduced binding interface (∼90 Å^2^), and markedly impaired docking score. In contrast, 2-*O*-desulfation had little effect, and 6-*O*-desulfation only moderately weakened the interaction in docking studies (Figure. [Fig fig1]I). These results suggest that *N*-sulfation is the key glycan determinant of charge-mediated Reelin-HS binding.

To validate the computational prediction, competition SPR (Figure 2A) was performed by using selectively desulfated heparin. Unmodified heparin (HP) fully blocked both FL-Reelin and Reelin-CTR binding to chip-immobilized heparin, whereas *N*-desulfated heparin (DeNS) showed much less inhibition, indicating the loss of binding of DeNS to Reelin in solution (Figure 2B-E). In contrast, 2-O- and 6-O-desulfated heparin inhibited binding almost as effectively as unmodified heparin in FL-Reelin (Figure 2B, C). A similar trend was observed for the Reelin-CTR results (Figure 2D, E). These results highlight the critical role of *N*-sulfation in mediating both FL-Reelin and Reelin-CTR interaction with HS, consistent with the computational prediction. In addition, the COLBOS mutation enhanced overall binding affinity but did not alter sulfation-site preferences for sulfation. Using competition SPR, we also examined the effect of heparin chain length on Reelin binding (Figure S4). The results indicated that the longer heparin chains exhibited stronger binding to Reelin.

**Figure 2:**
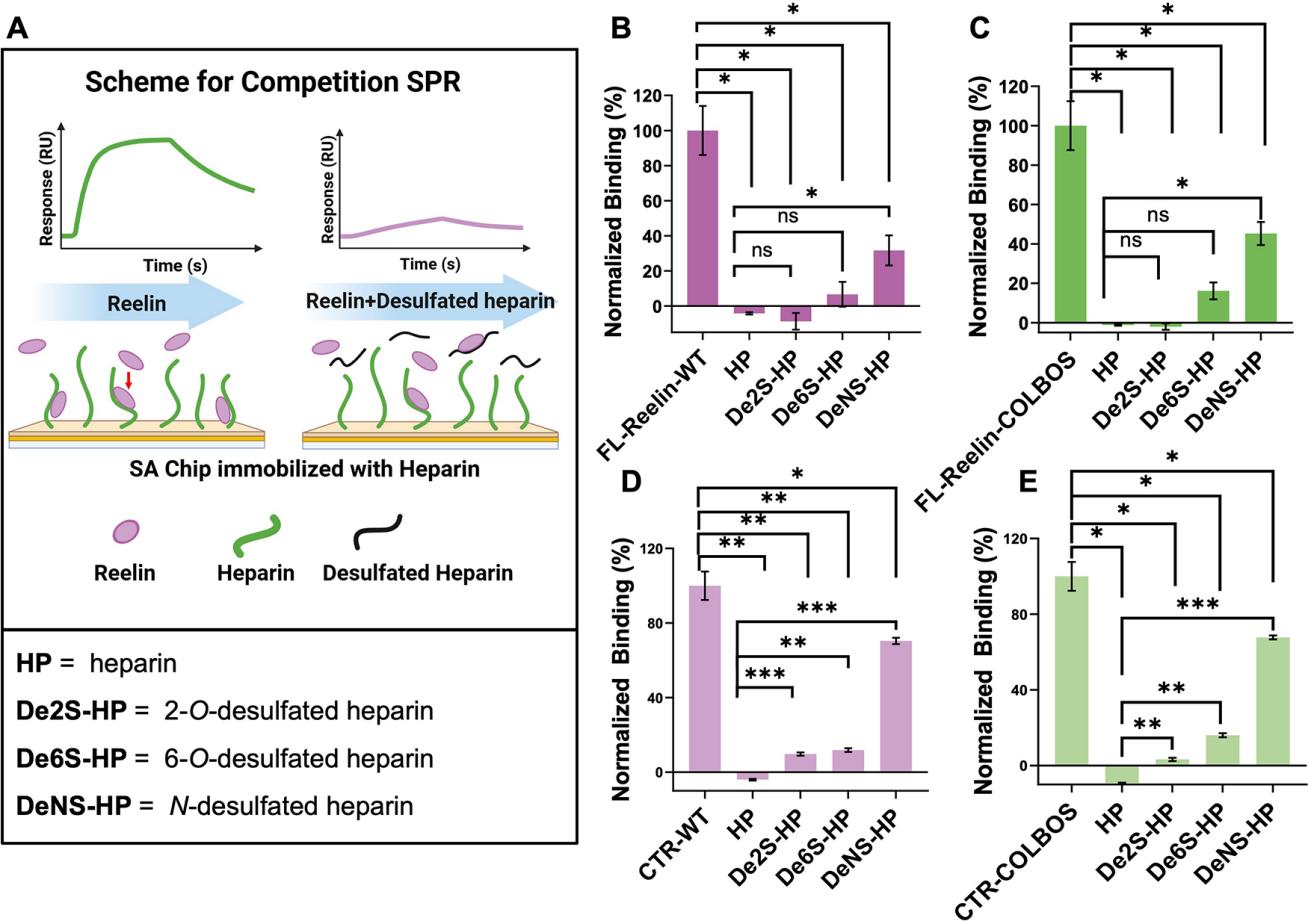
Competition SPR highlights the role of N-sulfation in the HP-Reelin interaction. (A) Schematic of the competition SPR assay. Biotinylated heparin was immobilized on an SA chip, and Reelin or its CTR was preincubated with heparin or desulfated heparin analogs before being flowed over the chip. The reduction in SPR response reflects the competition between heparin in solution vs heparin immobilized on the chip. (B–E) Normalized binding of FL-Reelin-WT (B), FL-Reelin-COLBOS (C), CTR-WT (D), and CTR-COLBOS (E) to immobilized heparin in the presence of unmodified heparin, *N*-desulfated heparin (DeNS-HP), 6-*O*-desulfated heparin (De6S-HP), or 2-*O*-desulfated heparin (De2S-HP). Statistical significance: *p < 0.05, **p < 0.01, ***p < 0.001.

We then used a glycan microarray of 96 chemically defined HS oligosaccharides^14^ (Figure S2) to validate the above results from desulfated heparin. Biotin-labeled Reelin-CTR bound to the HS-microarray was detected with fluorescence-labeled streptavidin. The results showed that Reelin binding intensity correlated strongly with chain length and sulfation level (Figure S3), consistent with competition SPR results (Figure S4). Among sulfation patterns, N-sulfation had the most pronounced positive effect: within oligosaccharides of the same chain length and disaccharide composition, the addition of each N-sulfate group markedly increased Reelin-CTR-WT binding (e.g., compound 11 vs compound 10), a trend reproduced across multiple oligosaccharide sets (Figure 3A, C). Reelin-CTR COLBOS displayed higher overall binding than WT but retained the same dependence on Nsulfation (Figure 3B, D). In contrast, 6S or 2S modifications showed no consistent effect on binding (Figure S3). To further confirm the role of N-sulfation in FL-Reelin binding, we compared two representative HS oligosaccharides, C67 and C68 (Figure 3C), which differ only by a single N-sulfate group. SPR analysis showed that C68 bound significantly more strongly to FL-Reelin, with FL-Reelin-WT exhibiting an ∼2.5-fold higher response to C68 than C67 (Figure 3E). Similar preferences were observed for COLBOS variants (Figure 3F), demonstrating that a single N-sulfation markedly enhances Reelin–HS interactions, highlighting the key role of N-sulfation.

**Figure 3:**
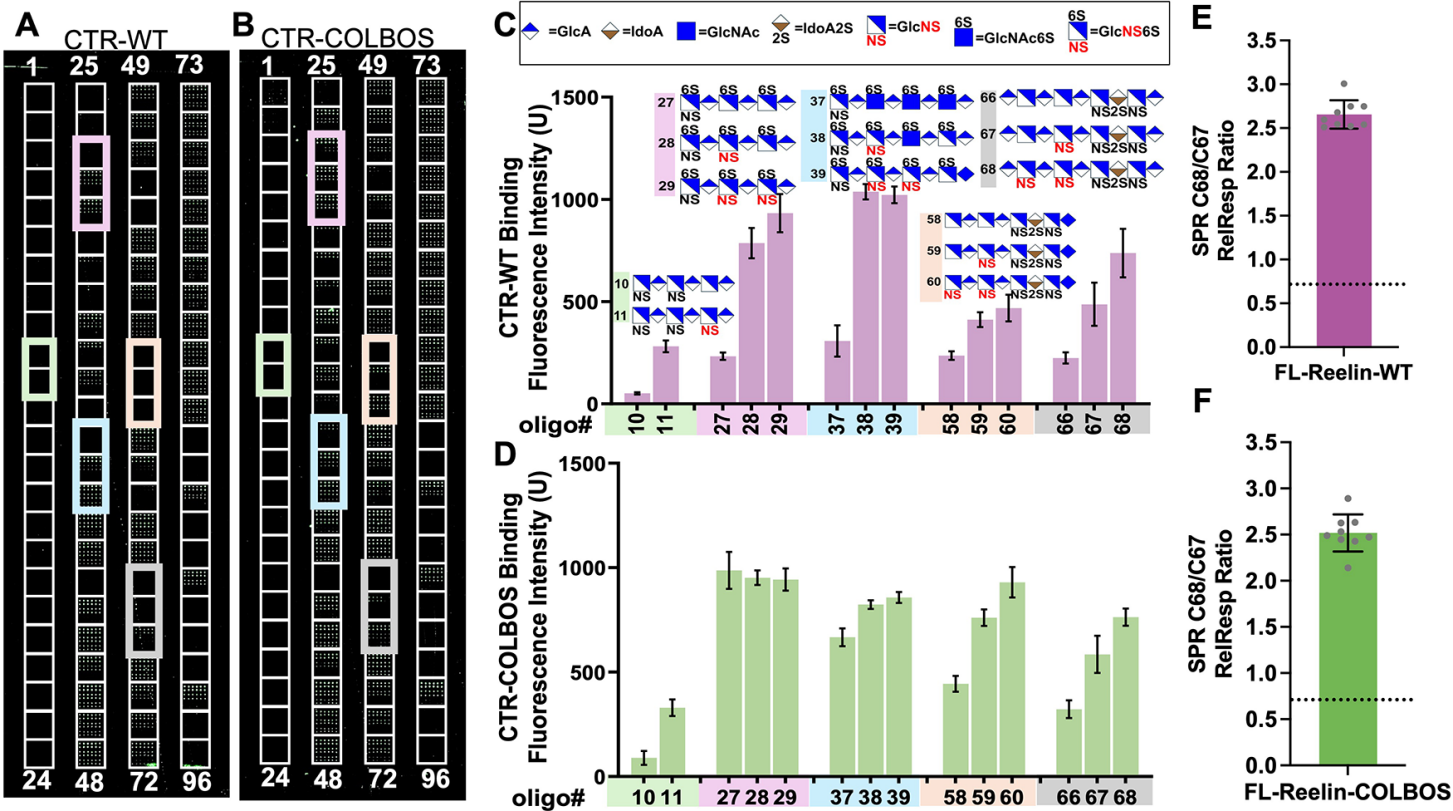
Glycan array analysis further confirms the importance of N-sulfation. (A, B) Representative images of the glycan microarray after incubation with Reelin CTR-WT (A) and Reelin CTR-COLBOS (B). NS-paired oligosaccharides, in which they differ only by single N-sulfations, are highlighted with distinct colored frames. (C, D) Fluorescence intensity representing the binding of Reelin-CTR-WT (C) and Reelin-CTR-COLBOS (D) to various low molecular weight heparan sulfate (LMWHs) with defined chemical structure, with varying sulfation patterns, disaccharide unit, and chain lengths. (E, F) SPR confirms N-sulfation in HS enhances full length Reelin binding for both WT and COLBOS variant. To show enhanced binding by N-sulfation, the SPR binding signals of Reelin-CTR-WT, Reelin-CTR-COLBOS, FL-Reelin-WT, and FL-Reelin-COLBOS were normalized to their binding signals to HS oligosaccharides 67. HS oligosaccharide 68 differs from 67 only by an additional *N*-sulfation group.

To evaluate the role of N-sulfation in Reelin-HS binding at the cellular level, we conducted cell surface binding assays. N-deacetylase/sulfotransferase 1 (Ndst1) is responsible for N-sulfation in heparan sulfate biosynthesis. We utilized wild-type (Ndst1f/f) mouse lung endothelial cells (MLECs) which have abundant HS on their cell surface, and Ndst1 knockout (Ndst1–/−) MLECs which show reduced levels of N-sulfation (by ∼40%), 6S (by ∼10%), and 2S (by ∼15%).^15^ The additional treatment with Heparinase I/III or heparin was included to examine the involvement of HS in binding of Reelin to the cell surface. Biotin-labeled Reelin-CTR (WT and COLBOS) bound to cell surfaces was detected using streptavidin-HRP. Heparinase I/III treatment or soluble heparin competition markedly reduced binding in Ndst1f/f cells (Figure 4A), confirming the critical role of HS in mediating Reelin’s interaction with the cell surface. The cell surface binding was further diminished in Ndst1–/– cells to ∼40% for WT and ∼30% for COLBOS, demonstrating that N-sulfation is essential for Reelin–HS interactions, consistent with SPR and glycan array results (Figure 4A).

**Figure 4:**
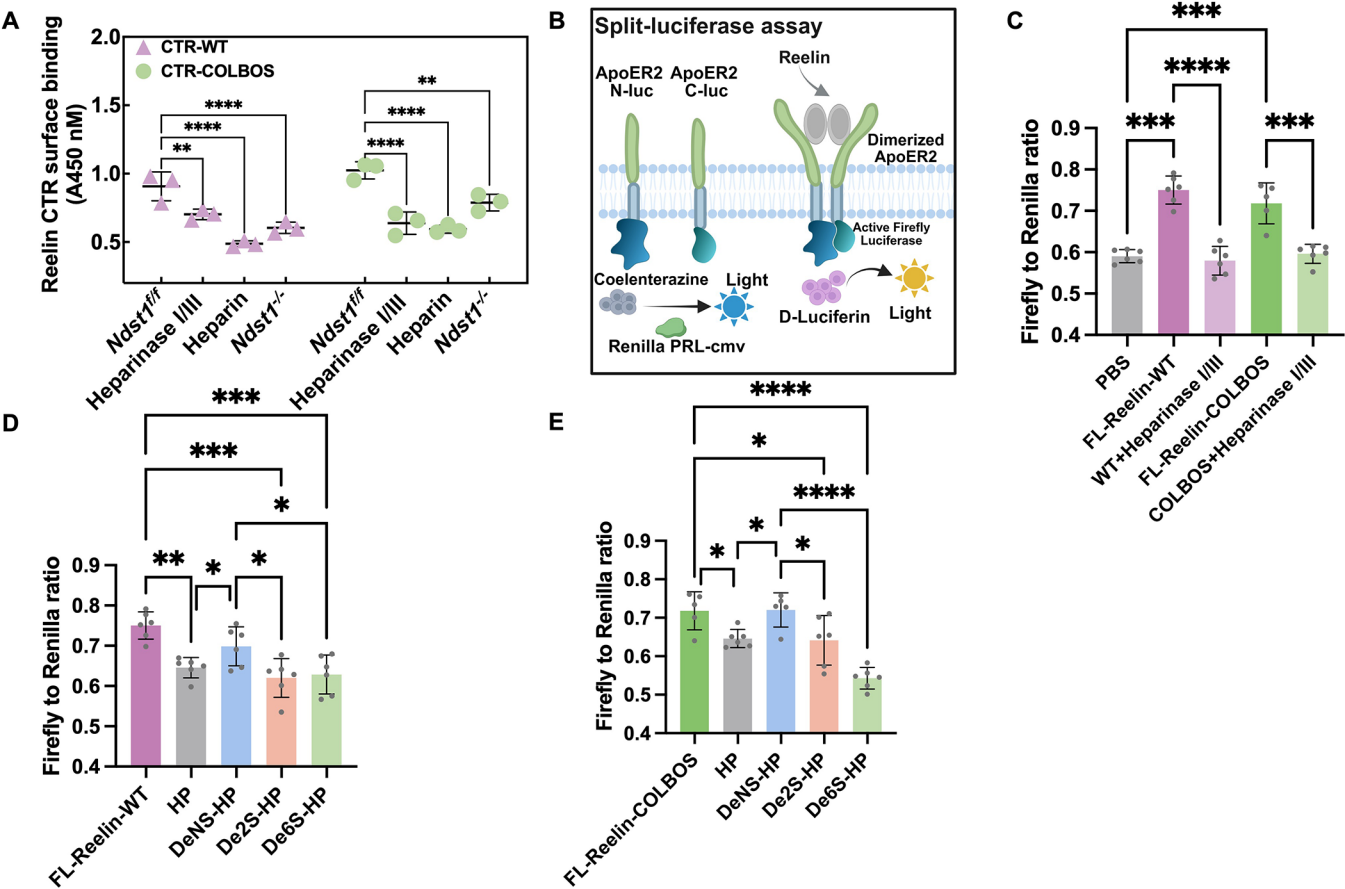
N-sulfation is critical for Reelin cell surface binding and Reelin-induced ApoER2 dimerization. (A) Cell surface binding of biotin-labeled Reelin-CTR-WT and Reelin-CTR-COLBOS. (B) Schematic representation of the split-luciferase assay. ApoER2 receptors labeled with N-luc and C-luc fragments generate luminescence upon dimerization triggered by Reelin binding. Firefly luciferase activity reflects dimerization, while Renilla luciferase serves as a normalization control. (C–E) Quantification of luminescence normalized to Renilla activity of different treatments on ApoER2 dimerization. Heparin (HP), N-desulfated heparin (DeNS-HP), 6-O-desulfated heparin (De6S-HP), 2-O-desulfated heparin (De2S-HP). Statistical significance: *p < 0.05, **p < 0.01, ***p < 0.001, ****p < 0.0001.

Reelin-mediated dimerization or higher-order multimers of ApoER2 are involved in the activation of the Reelin signaling pathway.^16,17^ To assess the role of HS in Reelin receptor activation, we employed a split-luciferase assay.^1^ HEK293T cells were transfected with plasmids encoding ApoER2-N-Luc, ApoER2-C-Luc, and Renilla Luciferase (as a control for transformation efficiency), where N-Luc and C-luc represent N- and C-terminal halves of firefly luciferase, respectively. Upon Reelin binding, ApoER2 will dimerize and reconstitute firefly luciferase activity (Figure 4B). In the luciferase assay, both FL-Reelin-WT and COLBOS significantly increased the Firefly/Renilla ratio relative to PBS, confirming receptor activation (Figure 4C). Heparinase I/III treatment degraded cell-surface HS and markedly reduced Reelin-induced ApoER2 dimerization in both WT and COLBOS, without affecting the Firefly/Renilla ratio (Figures 4C, S5). Exogenous heparin strongly inhibited ApoER2 dimerization, while desulfated derivatives (DeNS-HP, De2S-HP, and De6S-HP) showed differential effects. DeNS-HP had the weakest inhibition, highlighting the essential role of N-sulfation, whereas 2-O- and 6-O-sulfation were less critical, as De2S-HP and De6S-HP inhibited ApoER2 dimerization similarly to heparin (Figures 4D–E, S6). These results demonstrated that N-sulfation plays a crucial role in Reelin-HS binding at the cellular level.

Here, using SPR competition, glycan arrays, knockout cell lines, and luciferase assays, we show that HS acts as a glycan coreceptor in Reelin signaling activation, with its sulfation pattern serving as a key modulator of the Reelin–HS interaction (Figure 5). Both Reelin-WT and Reelin-COLBOS exhibit high-affinity binding to heparan sulfate and depend critically on N-sulfation for this interaction and downstream ApoER2 activation. The two proteins share similar overall binding profiles in surface plasmon resonance, glycan array, and cell-based assays, confirming that the COLBOS mutation does not alter the fundamental mechanism of Reelin–HS recognition. Nevertheless, the COLBOS variant shows a modest (∼1.5–2-fold) increase in overall affinity and a subtle shift in glycan preference, displaying stronger binding to HS structures containing fewer N-sulfation in both glycan array and cell-surface binding assays. H3447R substitution enhances the local positive charge density in the C-terminal domain, improving electrostatic complementarity with negatively charged HS motifs. Thus, our data suggest that the COLBOS mutation fine-tunes, rather than dramatically increases, Reelin–HS interactions, providing a plausible molecular basis for its reported neuroprotective effect. This work provides a potential basis for developing therapeutic strategies targeting Reelin signaling.

**Figure 5:**
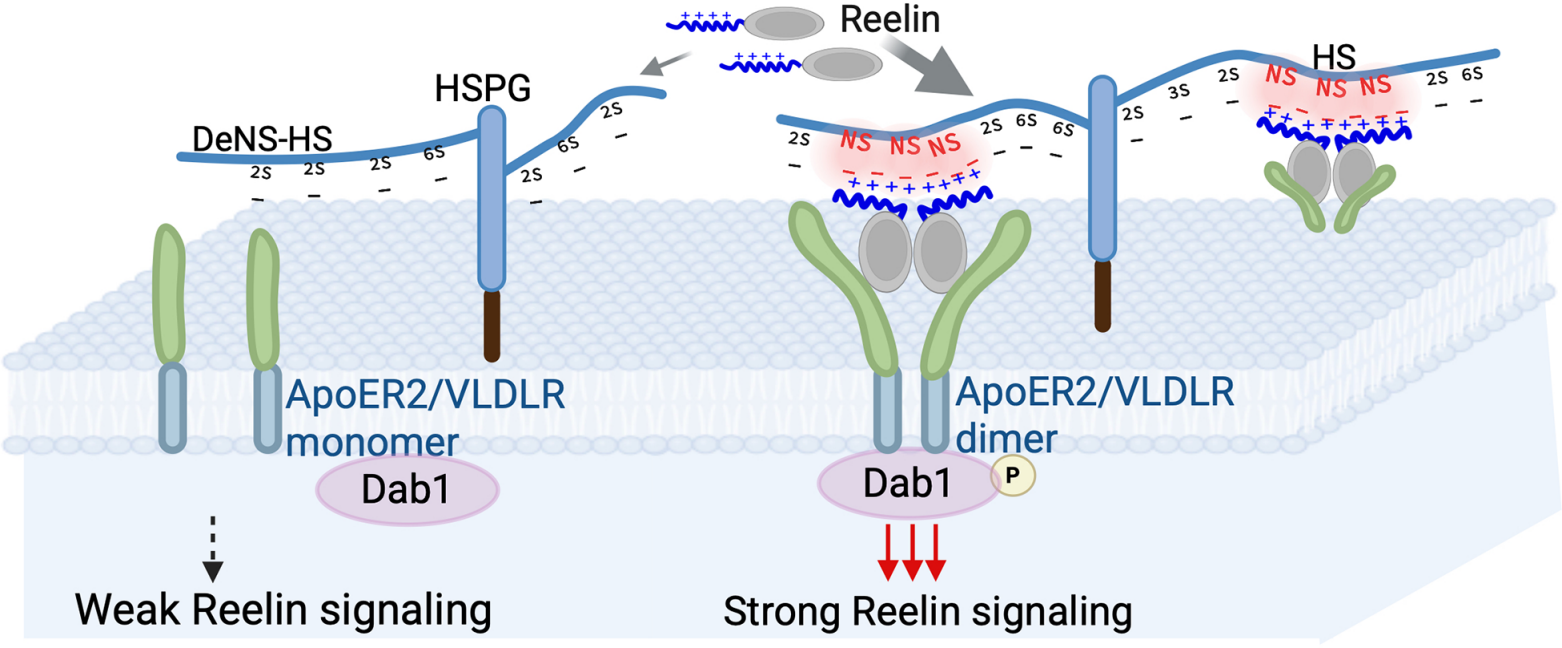
A model of Reelin-HS binding in Reelin signaling, where N-sulfation of HS (NS in Red) plays a key role by interacting with positive charges on Reelin. Strong Reelin-HS binding enhances Reelin signaling pathway by acting as a co-receptor. This activation leads to Dab1 phosphorylation and downstream effects.^2,4^

## Supplementary Material



## Notes

Figures S1–S6 and experimental procedures. (PDF)

## #

NMR of Large Assembles Group Institute of Structural Biology 71 Avenue des Martyrs, 38000 Grenoble, France

## References

[1] D’Arcangelo G., Nakajima K., Miyata T., Ogawa M., Mikoshiba K., Curran T. (1997). Reelin Is a Secreted
Glycoprotein Recognized by the
CR-50 Monoclonal Antibody. J. Neurosci..

[2] Hiesberger T., Trommsdorff M., Howell B. W., Goffinet A., Mumby M. C., Cooper J. A., Herz J. (1999). Direct binding of Reelin to VLDL
receptor and ApoE receptor 2 induces tyrosine phosphorylation of disabled-1
and modulates tau phosphorylation. Neuron.

[3] Bracher-Smith M., Leonenko G., Baker E., Crawford K., Graham A. C., Salih D. A., Howell B. W., Hardy J., Escott-Price V. (2022). Whole genome
analysis in APOE4 homozygotes identifies the DAB1-RELN pathway in
Alzheimer’s disease pathogenesis. Neurobiol
Aging.

[4] Joly-Amado A., Kulkarni N., Nash K. R. (2023). Reelin signaling in neurodevelopmental
disorders and neurodegenerative diseases. Brain
Sciences.

[5] Lopera F., Marino C., Chandrahas A. S., O’Hare M., Villalba-Moreno N. D., Aguillon D., Baena A., Sanchez J. S., Vila-Castelar C., Ramirez Gomez L. (2023). Resilience to autosomal
dominant Alzheimer’s disease in a Reelin-COLBOS heterozygous
man. Nature Medicine.

[6] Bandtlow C. E., Zimmermann D. R. (2000). Proteoglycans
in the developing brain: new conceptual
insights for old proteins. Physiol. Rev..

[7] Ozsan
McMillan I., Li J. P., Wang L. (2023). Heparan sulfate proteoglycan
in Alzheimer’s disease: aberrant expression and functions in
molecular pathways related to amyloid-beta metabolism. Am. J. Physiol Cell Physiol.

[8] Li J. P., Kusche-Gullberg M. (2016). Heparan sulfate:
biosynthesis, structure, and function. International
review of cell and molecular biology.

[9] Rauch J. N., Chen J. J., Sorum A. W., Miller G. M., Sharf T., See S. K., Hsieh-Wilson L. C., Kampmann M., Kosik K. S. (2018). Tau internalization
is regulated by 6-O sulfation on heparan sulfate proteoglycans (HSPGs). Sci. Rep..

[10] Mah D., Zhu Y., Su G., Zhao J., Canning A., Gibson J., Song X., Stancanelli E., Xu Y., Zhang F. (2023). Apolipoprotein
E Recognizes Alzheimer’s Disease Associated
3-O Sulfation of Heparan Sulfate. Angew. Chem.,
Int. Ed. Engl..

[11] Gama C. I., Tully S. E., Sotogaku N., Clark P. M., Rawat M., Vaidehi N., Goddard W. A., Nishi A., Hsieh-Wilson L. C. (2006). Sulfation
patterns of glycosaminoglycans encode molecular
recognition and activity. Nat. Chem. Biol..

[12] Reinhard A., Nürnberger T. (2017). Steady-state
and kinetics-based affinity determination
in effector-effector target interactions. Plant
Pattern Recognition Receptors: Methods and Protocols.

[13] Honorato R. V., Koukos P. I., Jiménez-García B., Tsaregorodtsev A., Verlato M., Giachetti A., Rosato A., Bonvin A. M. J. J. (2021). Structural biology in the clouds:
the WeNMR-EOSC ecosystem. Frontiers in molecular
biosciences.

[14] Li J., Cai C., Wang L., Yang C., Jiang H., Li M., Xu D., Li G., Li C., Yu G. (2019). Chemoenzymatic synthesis
of heparan sulfate mimetic glycopolymers and their interactions with
the receptor for advanced glycation end-product. ACS Macro Lett..

[15] Qiu H., Shi S., Yue J., Xin M., Nairn A. V., Lin L., Liu X., Li G., Archer-Hartmann S. A., Dela Rosa M. (2018). A mutant-cell
library for systematic analysis of heparan sulfate structure-function
relationships. Nat. Methods.

[16] Strasser V., Fasching D., Hauser C., Mayer H., Bock H. H., Hiesberger T., Herz J., Weeber E. J., Sweatt J. D., Pramatarova A. (2004). Receptor clustering
is involved in Reelin signaling. Molecular and
cellular biology.

[17] Li Q., Morrill N. K., Moerman-Herzog A. M., Barger S. W., Joly-Amado A., Peters M., Soueidan H., Diemler C., Prabhudeva S., Weeber E. J. (2023). Central repeat fragment of reelin leads to
active reelin intracellular signaling and rescues cognitive deficits
in a mouse model of reelin deficiency. Cell
Signal.

