# Supplementary material for: N‑Sulfated Heparan Sulfate Promotes Reelin Signaling as a Co-receptor
Source: J Am Chem Soc. 2025 Dec 8;147(51):46773–9. doi: 10.1021/jacs.5c15573 (PMC12781101; doi:10.1021/jacs.5c15573)
Supplement: Supplementary file 1 [file ja5c15573_si_001.pdf]

# N-Sulfated Heparan Sulfate Promotes Reelin Signaling as a Co-Receptor

*Lin Pan[1,2], Xuehong Song[3], Guowei Su[4], Lauren A Gandy[1,7], Biqin Fang[1,2], Mason Buttaci[1,2], James Gibson[1,2], Ke Xia[1], Fuming Zhang[1], Jian Liu[4,6], Lianchun Wang[3], Sally Temple[5], Chunyu Wang[1,2*]*

[1]Center for Biotechnology and Interdisciplinary Studies, Troy, NY12180 2Dept. of Biological Sciences; [2]Dept. of Chemistry and Chemical Biology, Rensselaer Polytechnic Institute, Troy NY 12180; [3] Dept. of Molecular Pharmacology and Physiology, Byrd Alzheimer's Center and Research Institute, Morsani College of Medicine, University of South Florida, Tampa, FL 33612; [4]Glycan Therapeutics, Raleigh, NC 27606; [5]Neural Stem Cell Institute, Albany, NY12208; [6]Division of Chemical Biology and Medicinal Chemistry, Eshelman School of Pharmacy, University of North Carolina, Chapel Hill, NC 27599. [7]Present address: Glycobiology, Cell Growth and Tissue Repair Research Unit (Gly-CRRET), Université Paris-est Créteil, Créteil, France.

* Corresponding author. E-mail address: wangc5@rpi.edu

## EXPERIMENTAL SECTION

### Expression of FL-Reelin in HEK293:

FL-Reelin was expressed using the Gibco Expi293 Expression System (Thermo Fisher Scientific, A14635) following the manufacturer's protocol. The expression plasmid for mouse FL-Reelin (pCrlM, Addgene Plasmid #122444) was used for transfection. The COLBOS variant of Reelin was generated by site directed mutagenesis on the pCrlM plasmid, performed by GenScript. Transfected cells were cultured in Expi293 medium under optimal conditions, and the supernatant containing Reelin protein was harvested for purification.

### Purification of FL- Reelin:

The harvested HEK293 culture supernatant was clarified by centrifugation and filtration through a 0.22 μm filter. The supernatant was first applied to a heparin affinity column (HiTrap Heparin HP, Cytiva, P:170701) equilibrated with Buffer A (20 mM Tris-HCl, pH 7.5). Bound protein was eluted using a linear gradient of Buffer B (20 mM Tris-HCl, pH 7.5, 2 M NaCl). The elevation was concentrated and subjected to further purification on a HiLoad 16/60 Superdex 200 size-exclusion chromatography column (Cytiva, P:171116601) pre-equilibrated with Buffer C (50 mM $NH_4HCO_3$). Fractions containing Reelin were collected, lyophilized, and analyzed for purity by SDS-PAGE and Western blot (anti-Reelin antibody G10, Abcam, ab78540). The reelin concentration was quantified using a known standard curve of bovine serum albumin. The purified Reelin protein was stored at -80°C for downstream analyses. Both wild-type and COLBOS variant proteins were purified following the same protocol.

### Reelin CTR overexpression and purification

Reelin CTR was expressed in *Escherichia coli* BL21 (DE3) (Thermo Fisher scientific, EC0114) cells. The plasmid encoding HIS-SUMO-CTR fusion protein was synthesized by GenScript. Transformed BL21 cells were grown in LB medium containing the appropriate antibiotic and induced with 0.5 mM IPTG at 37°C overnight to promote protein expression. The bacterial pellet was collected, resuspended in lysis buffer (50 mM Tris-HCl, pH 7.4, 200 mM NaCl, and 1 mM PMSF), and lysed by sonication. The lysate was clarified by centrifugation at 12,000 × g for 30 minutes at 4°C, and the supernatant was loaded onto a nickel-affinity column (HisTrap HP, Cytiva, P: 17524801) pre-equilibrated with lysis buffer. The HIS-SUMO-CTR fusion protein was eluted using an imidazole gradient (0–500 mM). The eluted protein was treated with ULP1 protease to remove the HIS-SUMO tag, and the reaction mixture was subsequently subjected to high-performance liquid chromatography (HPLC) using a C18 column (ZORBAX 300SB-C18 PrepHT, Agilent, 5 21.2 × 250 mm, 7 μm). The column was equilibrated with solvent A (0.1% trifluoroacetic acid in water), and the CTR was eluted with a gradient of solvent B (0.1% trifluoroacetic acid in acetonitrile). The final purified Reelin CTR was collected, lyophilized, and stored at -20°C. The purity and integrity of the purified CTR were confirmed by SDS-PAGE and Western blot analysis using Anti-Reelin C-Term Antibody (clone 12C10) (Sigma-Aldrich,

MABN2428). The purified CTR was used for downstream experiments.

### Structure-guided docking of Reelin and HS tetrasaccharides

Structure-guided docking was performed using HADDOCK 2.4[1]. The Reelin C-terminal region (CTR) was extracted from PDB: 8G21. A fully sulfated canonical HS tetrasaccharide was modeled, along with desulfated variants (deNS, de2S, de6S) lacking specific N-, 2-O-, or 6-O-sulfate groups, respectively. Docking was performed using the guru interface in blind docking mode without defining active residues. Center-of-mass restraints were applied to promote molecular contact during initial sampling. A total of 10,000 rigid-body docking models were generated, followed by semi-flexible refinement of the top 400 structures and final refinement in water for 400 models. Final scoring and cluster ranking were based on the top 400 refined structures, using HADDOCK's standard weighted energy function, which includes van der Waals, electrostatic, desolvation, and restraint violation terms. The best-scoring cluster was selected based on HADDOCK score and cluster size. Molecular graphics and interface visualizations were prepared using MOE 2022.

### Preparation of Biotin-Labeled Reelin CTR

Reelin CTR was biotinylated using the EZ-Link Sulfo-NHS-Biotin reagent (Thermo Scientific, P:21217). Briefly, purified Reelin CTR was incubated with Sulfo-NHS-Biotin in a molar ratio of 1:20 in labeling buffer (50 mM phosphate buffer, pH 7.5, 150 mM NaCl) at room temperature for 30 minutes with gentle agitation. The reaction mixture was then subjected to a C18 column (ZORBAX 300SB-C18 PrepHT, Agilent, 5 21.2 × 250 mm, 7 μm) to remove the unconjugated biotin. Fractions containing the labeled CTR were collected, lyophilized, and stored at -20°C until use.

### Characterization of Reelin-HS interaction by SPR assay

The HS biochip was prepared as previously described[2]. Briefly, the biotinylated heparin was immobilized onto a SA chip (Cytiva, P:29699621). Unbound sites on the chip surface were blocked with 1 M ethanolamine (pH 8.5) to prevent nonspecific binding. The chip was equilibrated with running buffer (HBS supplemented with 0.005% Tween-20) before binding analyses.

Binding affinity between Reelin and heparin was assessed using a Biacore T200 SPR from-Cytiva (Uppsala, Sweden). Purified Reelin proteins (both full-length and CTR, including WT and COLBOS variants) were diluted in running buffer and injected over the heparin-coated chip at various concentrations. The binding response was recorded in real time and analyzed to determine association and dissociation rates. The data was analyzed with Biacore T200 Evaluation software 3.2 (Uppsala, Sweden).

### SPR Competition Assay

To evaluate the impact of HS modifications on Reelin binding, a competition assay was performed.

As previously described[3], purified Reelin was pre-incubated with free HS or modified HS oligosaccharides (e.g., *N*-desulfated, 6-*O*-desulfated, or 2-*O*-desulfated, final concentrations 1 µM) (Iduron, DSH003, DSH002, DSH001) at room temperature. The pre-incubated mixture was then injected over the HS-coated chip. The binding response was measured and compared to that of Reelin alone to determine the inhibitory effects of free HS or modified oligosaccharides.

**The binding preference of Reelin to HS using glycan microarray assay[4, 5]**

The glycan microarray analysis was performed as previously described[4, 6]. 96 heparan sulfate (HS) oligosaccharides were immobilized onto a microarray chip at three concentrations (50 µM, 25 µM, and 12.5 µM). Biotin-labeled Reelin-CTR (30 µg/mL in PBS) was introduced onto the chip and incubated for 1 hour at room temperature. After incubation, unbound Biotin-labeled Reelin-CTR was washed off the chip twice with PBS then the chip was incubated with fluorescence-labeled streptavidin. After wash, the chip was then scanned using a GenePix 4300 scanner (Molecular Dynamics, Caesarea, Israel) at a resolution of 5 µm, with excitation at 488 nm to detect bound biotinylated protein. Array images were analyzed with GenePix Pro 7.2.29.002 software, which automatically identified and quantified the mean fluorescence intensities of 24 replicates for each oligosaccharide concentration. All experiments were conducted in triplicate to ensure reproducibility.

**Cell surface binding assay**

Wild-type ($Ndst1^{f/f}$) and its daughter *Ndst1* knockout ($Ndst1^{-/-}$) mouse lung endothelial cell lines were used to evaluate the role of *N*-sulfation in Reelin-CTR cell surface binding. Following the established protocol[7], MLECs were seeded in 96-well plates with DMEM supplemented with 10% FBS, 100 U/mL penicillin, and 100 µg/mL streptomycin, then incubated overnight at 37°C with 5% $CO_2$. The next day, the cells were washed with DPBS, fixed with 4% paraformaldehyde (PFA) for 15 minutes at room temperature, rewashed, and blocked with DPBS containing 1% BSA for 90 minutes. Biotinylated Reelin-CTR (8 µg/mL) was added and incubated for 90 minutes at room temperature. After incubation, the cells were washed, and Streptavidin-HRP (1:2000 dilution in DPBS with 1% BSA) was applied for 30 minutes. Bound Reelin-CTR was detected using the Ultra TMB-ELISA kit (Thermo Scientific, 34028) according to the manufacturer's instructions. All experiments were performed in triplicate, and HRP activity was measured to quantify cell surface binding.

**Split Luciferase Assay**

Plasmids encoding ApoER2 fused with N-terminal luciferase (ApoER2-N-luc) and C-terminal luciferase (ApoER2-C-luc) fragments were constructed as previously described[8]. HEK293T cells were seeded in 96-well plates at a density of $1 \times 10^5$ cells per well and transiently transfected with plasmids encoding ApoER2-N-luc and ApoER2-C-luc using Lipofectamine 2000 (Thermo Fisher Scientific, P: 11668027) according to the manufacturer's protocol. *Renilla* luciferase plasmid

(PRL-cmv, Promega, E6931) was co-transfected as a normalization control.

After 24 hours of transfection, cells were treated with 100 nM of purified Reelin proteins (FL-Reelin-WT, FL-Reelin-COLBOS) in the presence or absence of heparin, Heparinase I/III, *N*-desulfated, 6-*O*-desulfated, or 2-*O*-desulfated heparin (3 μM). The cells were incubated with the treatments for 1 h at 37°C. Firefly luciferase activity, reflecting the dimerization of ApoER2, and *Renilla* luciferase activity, serving as the normalization control, were measured using the Dual-Glo Luciferase Assay System (Promega, N1610). Firefly and *Renilla* luminescence were recorded using a TECAN Spark® microplate reader. The data was analyzed by calculating the ratio of firefly/*Renilla* luminance for each well.

**Statistics**

Data obtained from experiments repeated at least three times was represented as mean ± SD unless specifically mentioned. Data were analyzed by two-tailed Student's T test or two-way ANOVA with Tukey's multiple comparisons test. p values less than 0.05 were considered statistically significant. All graphs and all statistical tests were generated using Graph Prism 8.0.2 software and Biorender.

## Supplementary figures

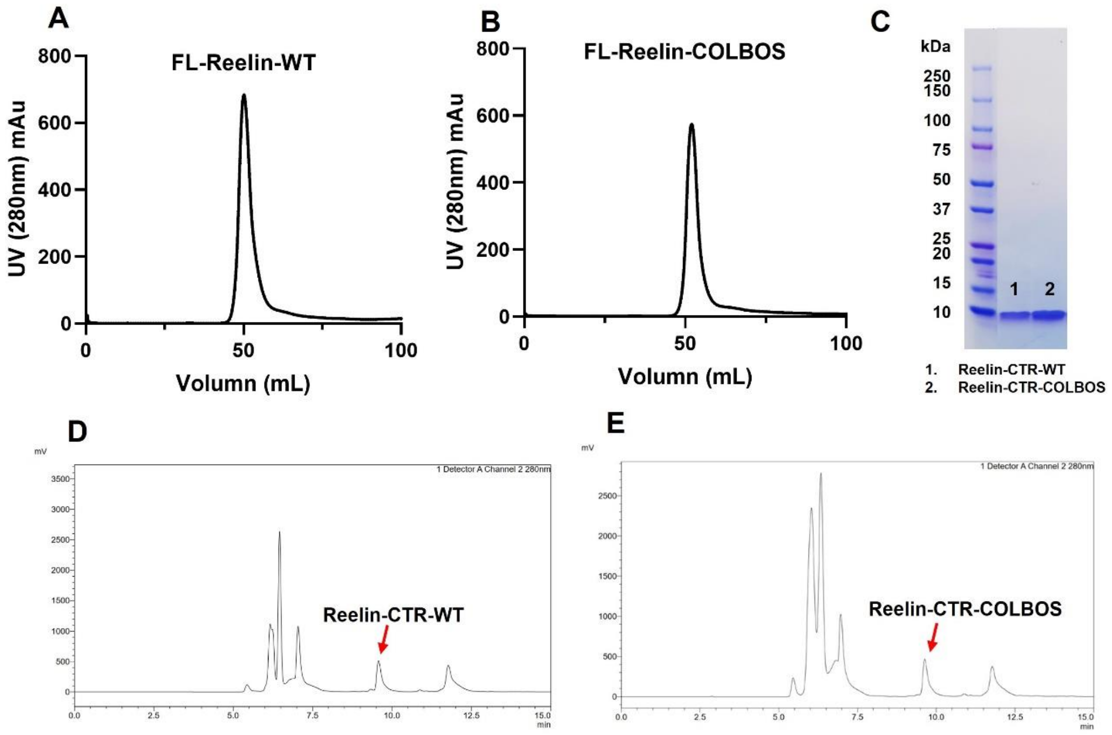


**Figure S1.** Purification of FL-Reelin and Reelin-CTR.
(A, B) Size-exclusion chromatography profiles of FL-Reelin-WT (A) and FL-Reelin-COLBOS (B) using a HiLoad 16/60 Superdex 200 column. Both constructs eluted as a single major peak at approximately 50 mL, indicating successful purification and homogeneity. (C) SDS-PAGE image of Reelin-CTR. (D, E) High-performance liquid chromatography (HPLC) profiles of Reelin-CTR-WT (D) and Reelin-CTR-COLBOS (E) using a C18 column. The red arrows highlight the elution peaks corresponding to Reelin-CTR-WT and Reelin-CTR-COLBOS, respectively.

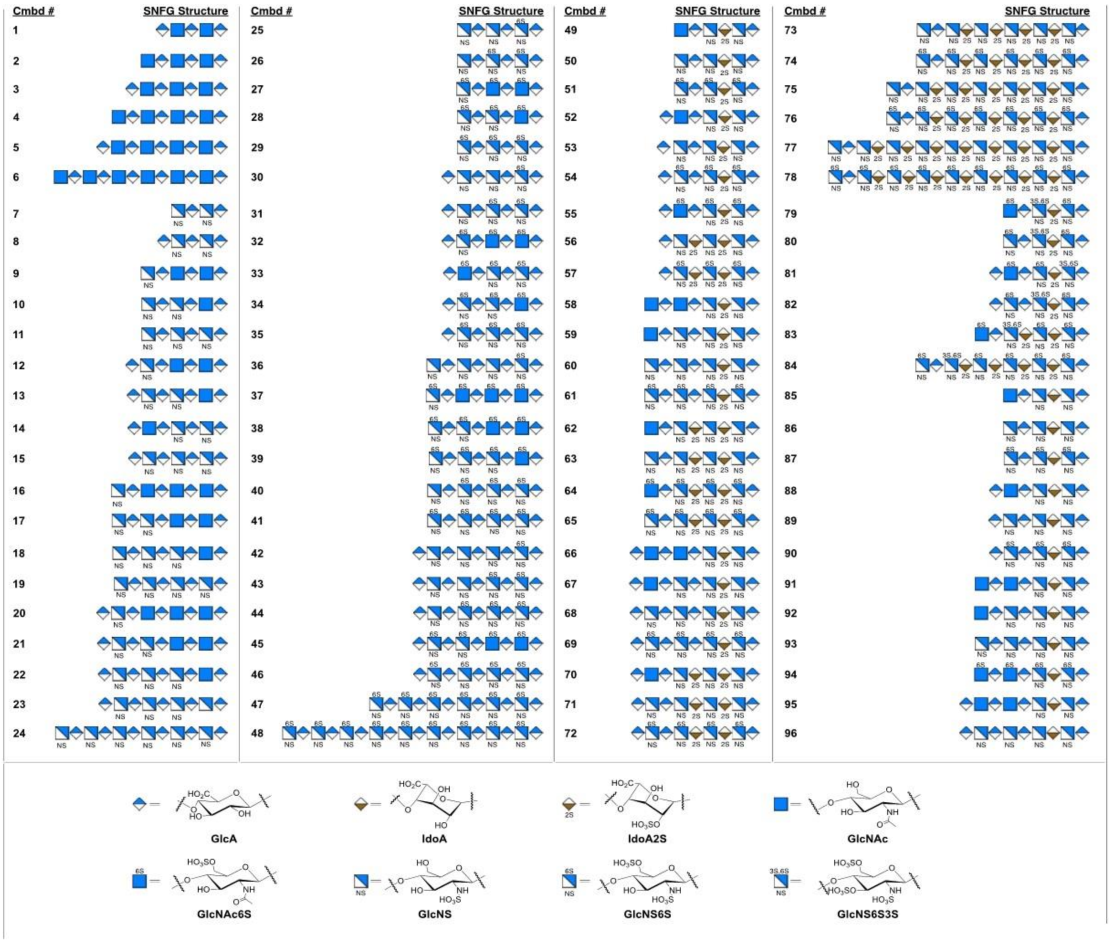


**Figure S2.** Structures of heparan sulfate (HS) oligosaccharides in the glycan microarray. The glycan microarray contains 96 chemically defined HS oligosaccharides with varying sulfation patterns and lengths. Each structure is represented using the Symbol Nomenclature for Glycans (SNFG) system, where specific monosaccharides and their modifications (e.g., sulfation) are color-coded and annotated. The bottom panel provides the legend for monosaccharide symbols and their chemical modifications, including GlcA, IdoA, GlcNAc6S, GlcNS, and others. This well-characterized array serves as a versatile tool for investigating protein-HS interactions

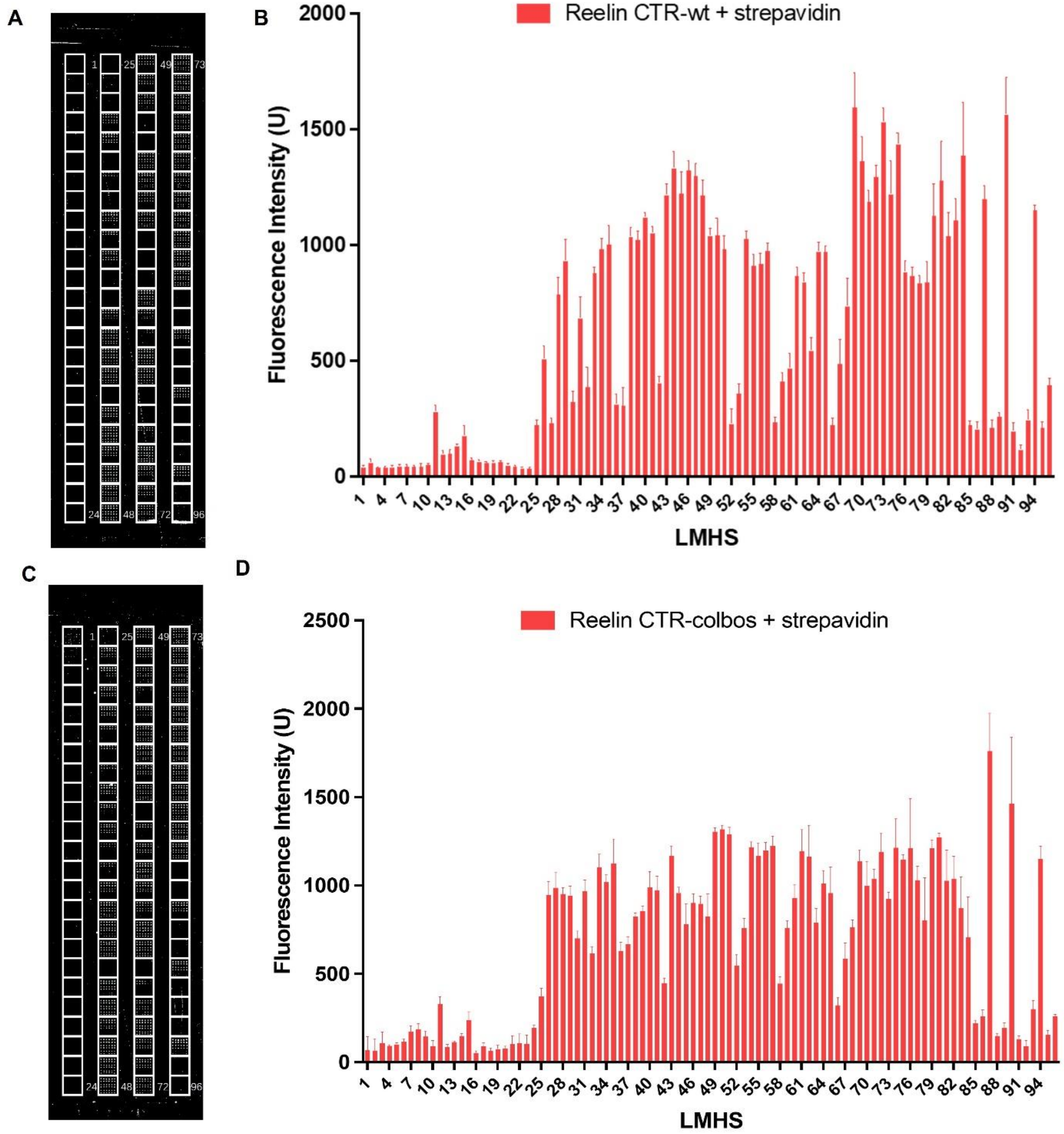


**Figure S3.** Glycan microarray analysis of Reelin CTR binding to heparan sulfate (HS) oligosaccharides. (A, C) Representative images of the glycan microarray after incubation with Reelin CTR-WT (A) and Reelin CTR-COLBOS (C) in the presence of streptavidin. Each spot corresponds to one of the 96 HS oligosaccharides.

(B, D) Quantification of fluorescence intensity (U) for Reelin CTR-WT (B) and Reelin CTR-COLBOS (D) binding to the glycan microarray. The x-axis represents the library of HS oligosaccharides (LMHS), and the y-axis shows the fluorescence intensity. Error bars represent standard deviation from replicates.

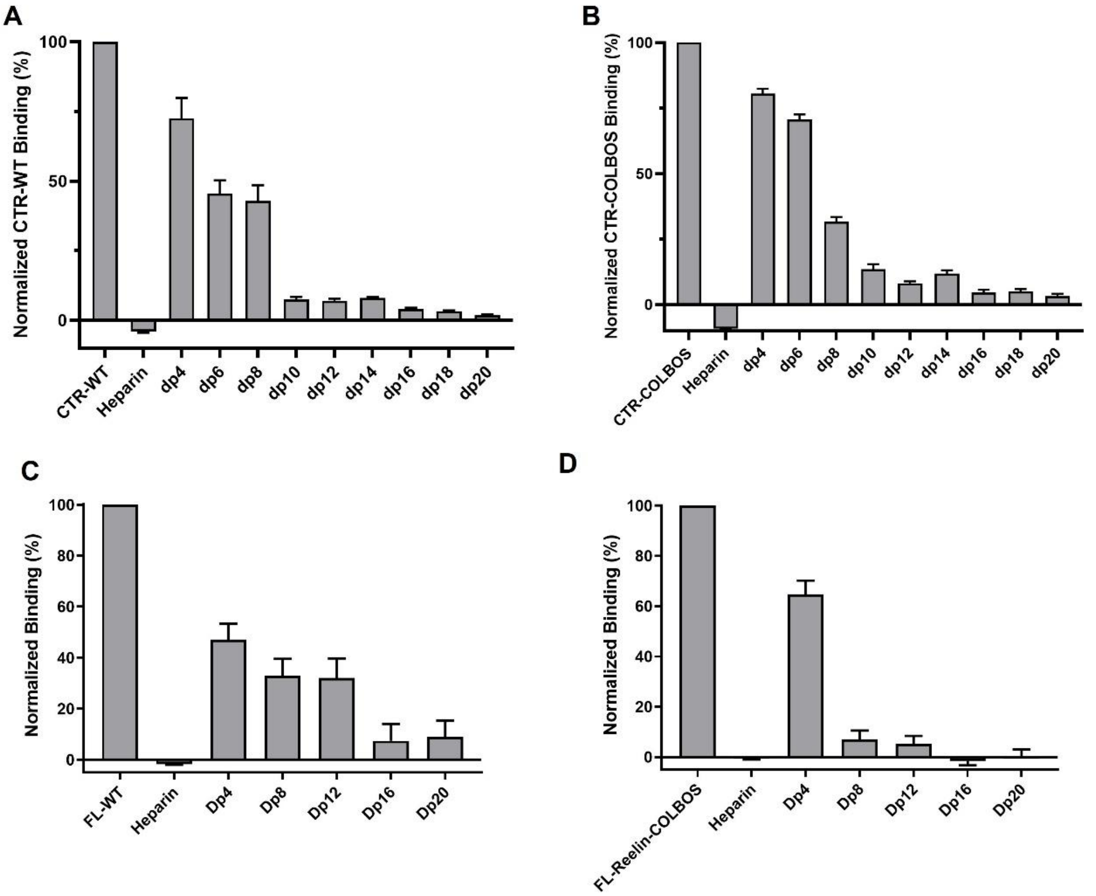


**Figure S4.** SPR competition analysis of Reelin CTR and FL-Reelin binding to heparan sulfate (HS) oligosaccharides of varying chain lengths. (A, B) Normalized binding of CTR-WT (A) and CTR-COLBOS (B) to immobilized heparin in the presence of free HS oligosaccharides with defined chain lengths (dp4–dp20). (C, D) Normalized binding of FL-Reelin-WT (C) and FL-Reelin-COLBOS (D) to immobilize heparin under the same competitive conditions. All data are normalized to the binding level without competitors (100%). Error bars represent the standard deviation of at three independent experiments.

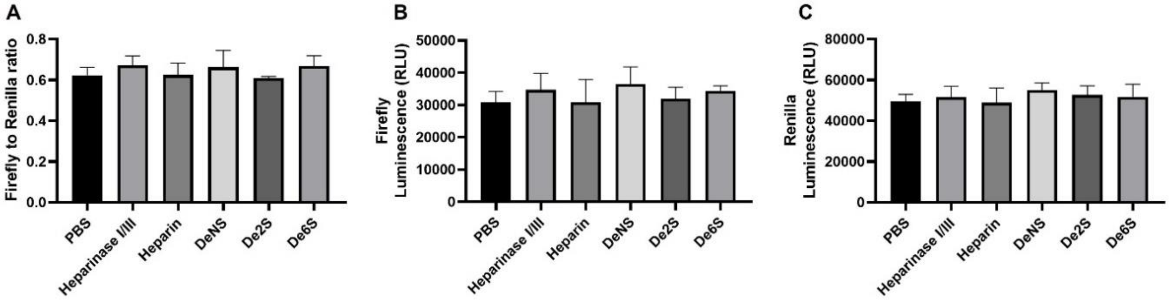


**Figure S5.** Negative control experiments to show that there is no effect for heparinase, heparin, DeNS-HP, De2S-HP, or De6S-HP on split-luciferase assay. (A) Firefly to Renilla ratio (B) Firely luminescence and (C) *Renilla* luminescence.

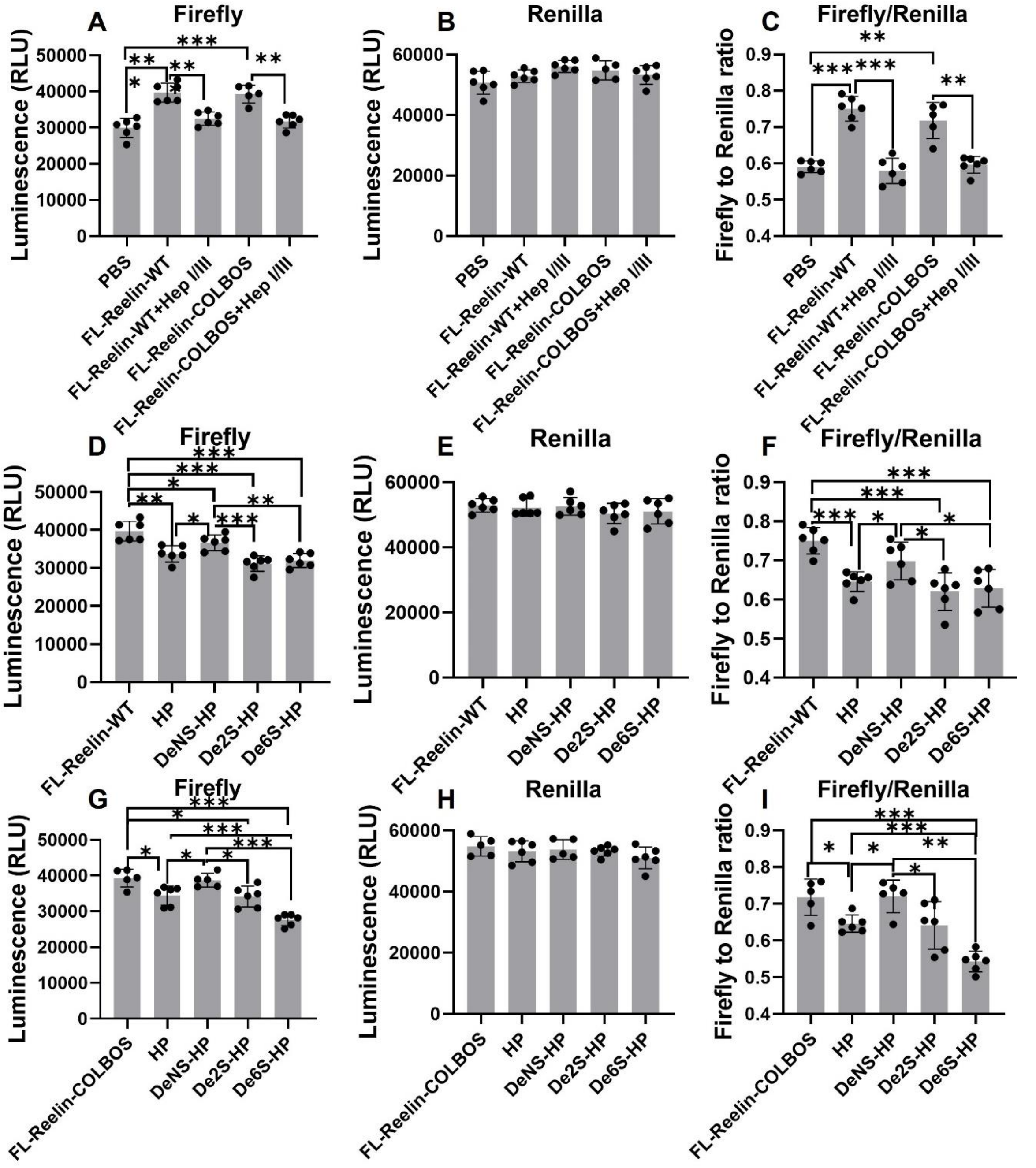


**Figure S6.** *N*-sulfation is critical for Reelin-induced ApoER2 dimerization as demonstrated by the split-luciferase assay. (A, D, G) Firefly luciferase. (B, E, H) *Renilla* luciferase. (C, F, I) Quantification of luminescence normalized to *Renilla* activity of different treatments on ApoER2 dimerization. Heparinase I/III, Heparin (HP), N-desulfated heparin (DeNS-HP), 6-O-desulfated heparin (De6S-HP), 2-O-desulfated heparin (De2S-HP). Statistical significance: *p < 0.05, **p < 0.01, ***p < 0.001.

## References

(1) Honorato, R. V.; Koukos, P. I.; Jiménez-García, B.; Tsaregorodtsev, A.; Verlato, M.; Giachetti, A.; Rosato, A.; Bonvin, A. M. J. J. Structural biology in the clouds: the WeNMR-EOSC ecosystem. *Frontiers in molecular biosciences* **2021**, *8*, 729513. Honorato, R. V.; Trellet, M. E.; Jiménez-García, B.; Schaarschmidt, J. J.; Giulini, M.; Reys, V.; Koukos, P. I.; Rodrigues, J. P.; Karaca, E.; van Zundert, G. C. P. The HADDOCK2. 4 web server for integrative modeling of biomolecular complexes. *Nature protocols* **2024**, *19* (11), 3219–3241.

(2) Kwon, P. S.; Oh, H.; Kwon, S.-J.; Jin, W.; Zhang, F.; Fraser, K.; Hong, J. J.; Linhardt, R. J.; Dordick, J. S. Sulfated polysaccharides effectively inhibit SARS-CoV-2 in vitro. *Cell discovery* **2020**, *6* (1), 50.

(3) Mah, D.; Zhu, Y.; Su, G.; Zhao, J.; Canning, A.; Gibson, J.; Song, X.; Stancanelli, E.; Xu, Y.; Zhang, F.; et al. Apolipoprotein E Recognizes Alzheimer's Disease Associated 3-O Sulfation of Heparan Sulfate. *Angew Chem Int Ed Engl* **2023**, *62* (23), e202212636.

(4) Gandy, L. A.; Canning, A. J.; Lou, H.; Xia, K.; He, P.; Su, G.; Cairns, T.; Liu, J.; Zhang, F.; Linhardt, R. J. Molecular determinants of the interaction between HSV-1 glycoprotein D and heparan sulfate. *Frontiers in Molecular Biosciences* **2022**, *9*, 1043713.

(5) Li, J.; Cai, C.; Wang, L.; Yang, C.; Jiang, H.; Li, M.; Xu, D.; Li, G.; Li, C.; Yu, G. Chemoenzymatic synthesis of heparan sulfate mimetic glycopolymers and their interactions with the receptor for advanced glycation end-product. *ACS Macro Letters* **2019**, *8* (12), 1570–1574.

(6) Zhao, J.; Zhu, Y.; Song, X.; Xiao, Y.; Su, G.; Liu, X.; Wang, Z.; Xu, Y.; Liu, J.; Eliezer, D.; et al. 3-O-Sulfation of Heparan Sulfate Enhances Tau Interaction and Cellular Uptake. *Angew Chem Int Ed Engl* **2020**, *59* (5), 1818–1827.

(7) Qiu, H.; Shi, S.; Yue, J.; Xin, M.; Nairn, A. V.; Lin, L.; Liu, X.; Li, G.; Archer-Hartmann, S. A.; Dela Rosa, M. A mutant-cell library for systematic analysis of heparan sulfate structure–function relationships. *Nature methods* **2018**, *15* (11), 889–899.

(8) Li, Q.; Morrill, N. K.; Moerman-Herzog, A. M.; Barger, S. W.; Joly-Amado, A.; Peters, M.; Soueidan, H.; Diemler, C.; Prabhudeva, S.; Weeber, E. J.; et al. Central repeat fragment of reelin leads to active reelin intracellular signaling and rescues cognitive deficits in a mouse model of reelin deficiency. *Cell Signal* **2023**, *109*, 110763.
